# Why Frailty Should Be Assessed in Older Adults Affected by Leprosy and How the Use of Smartphones Could Enhance This Health Monitoring

**DOI:** 10.2196/55963

**Published:** 2026-08-06

**Authors:** Luzielma Macedo Gloria, Josafá Gonçalves Barreto, João Sérgio de Sousa Oliveira, Areolino Pena Matos, Bianca Callegari, Givago Souza

**Affiliations:** 1 Federal University of Pará, Generalíssimo Deodoro Avenue 92, Belém, 66055240, Brazil, 55 91982653131; 2 University of Pará State, Belém, Brazil

**Keywords:** leprosy, digital health, smartphones, balance control, mobility assessment, digital public health, frailty, sensors, mHealth, gerontology, older adults, aging, monitoring, motor function, technology use, health tools

## Abstract

Leprosy is endemic in many low-income countries, affecting mainly the poorest communities. The disease can lead to sensorimotor functional losses that can predispose patients to reduced independence. In older adults, these losses associated with leprosy are added to those already resulting from natural aging and associated comorbidities, which can contribute to the clinical picture of frailty syndrome in these older adults. Early detection of frailty can reduce several serious consequences for these individuals, preventing loss of quality of life and financial costs to patients and the health care system. Sensors built into smartphones have been proposed as low-cost, and highly sensitive tools for detecting motor loss and could help monitor the risk of some frailty features in older people affected by leprosy.

## Introduction

Population aging affects all countries today. While high-income nations have already plateaued, transitional and low-income countries are still undergoing the process [1]. Aging causes physiological and structural changes, resulting in some degree of loss of function and independence [2]. Additionally, various diseases can result in physical impairment in old people [3].

In certain circumstances, older adults may experience physical, psychological, and social challenges that reduce their ability to tolerate stressors and cause a cumulative decline in organic functions, making them susceptible to falls and potential negative consequences, including death [4]. The term used to describe this geriatric condition is frailty or frailty syndrome, which is characterized by three of five phenotypes: weakness, low level of physical activity, unintentional weight loss, slow gait speed, and exhaustion [5].

Transitional countries, such as Brazil and India have an impoverished population affected by numerous endemic diseases. Although frailty syndrome may be common in these populations, it is largely unrelated to these endemic diseases. However, leprosy can be a significant comorbidity factor, which, when combined with frailty, can greatly increase the risk of physical disabilities [6].

In regions in which leprosy is endemic, balance control and mobility are not adequately assessed in primary care services due to the high cost and limited availability of gold-standard assessment tools, which are primarily found in research or referral centers. Evaluation of balance or mobility is often conducted through the use of scales or low-cost clinical tests, which may lack the sensitivity necessary to identify early fall risks [7-9]. New options using portable digital technologies have been suggested and validated for conducting this kind of motor evaluation [10,11].

In this context, we suggest discussing the significance of evaluating frailty syndrome in older people with leprosy as a crucial public health policy. Additionally, we propose smartphones as a digital health intervention that could improve the monitoring of the risk of falls in the overlooked population of older adults with leprosy.

## The Assessment of Frailty in Leprosy

Leprosy, caused by the bacterium *Mycobacterium leprae*, or less frequently by the *Mycobacterium lepromatosis*, is an infectious disease that can result in neuropathies compromising proper somatosensory, visual, and vestibular system functioning, as well as muscle strength [12-14]. Although these functions are altered, balance assessment is not included in the standardized protocols established by the World Health Organization (WHO) [15], which serves as the foundation for national protocols in countries like Brazil.

The WHO disability grading system recommends assessing tactile sensitivity, muscle strength, and vision [15]. However, despite vision, somesthesia, and muscular activity contributing to balance control, individualized assessment of each of these functions does not provide a comprehensive understanding of balance control. Furthermore, evaluations of the vestibular system or other somatosensory modalities including temperature, pain, and proprioception, are not considered. It is crucial to note that there is no direct evaluation of mobility or balance in patients with leprosy.

Considering the widespread lack of assessment for both balance and mobility in patients with leprosy of all age groups, the issue becomes particularly concerning in older adults [6]. This population already experiences natural functional declines that are associated with physiological aging, as well as other comorbidities that are more prevalent as individuals age. These comorbidities, such as diabetes, hypertension, and osteoporosis, add to the functional impairment experienced by older adults.

Assessing frailty in older individuals with leprosy holds paramount importance for several reasons. The identification of frailty in older adults with leprosy would enable the timely detection of potential complications (increased risk of disability, delayed wound healing and ulcer development, increased falls, psychological complications, reduce quality of life, etc), facilitate physical and psychological interventions during milder clinical conditions, and provide health managers with valuable insights, thereby contributing to the formulation of new health policies and health care measures [16,17].

Since frailty specifically causes a decrease in muscle mass and strength, which can significantly affect balance control and mobility in older adults with leprosy, monitoring these functions may aid health managers in identifying those who may be at the risk of developing frailty syndrome.

## Tracking Frailty Using Smartphones

There is a growing interest in using smartphones to track frailty in older adult populations, and several studies have explored their potential. Most of the apps used to track fragility used applications with forms to be filled with clinical descriptions of the patients following the criteria to classify the Fried’s frailty phenotype [18,19]. Other apps have validated the use of sensors from smartphones (cameras and/or inertial sensors) to quantify functional activities that would be associated with the features of fragility [20,21].

## Evaluation of Movement as a Potential Indicator of Functional Losses Related to Frailty

The high cost and limited accessibility of gold-standard movement analysis methods, like force platforms, electromyography, and video capture systems, pose significant barriers among poorer populations. These instruments require highly sensitive sensors and robust construction to ensure accuracy and regular, specialized calibration to maintain reliability, and are accompanied by advanced analytics software, thereby adding to the overall cost. These systems often require controlled environments, reliable power supplies, and ample physical space, which may be unavailable in resource-limited areas. Skilled professionals are necessary to operate and interpret the data from these systems, but such expertise is often lacking in underserved regions. These are often only found in specialized research laboratories, hospitals, or sports centers, limiting access for general populations.

However, smartphones with a multitude of built-in sensors have recently emerged, offering validated low-cost alternatives for a range of motor tests. These devices have the potential to provide high-precision assessments in primary care settings [22].

Specifically, for the evaluation of balance control and mobility, various proposals have demonstrated that the inertial sensors, namely the accelerometer and gyroscope, present in smartphones can extract numerous characteristics that describe these functions [23-25].

For static balance assessment, accelerations in the anteroposterior and mediolateral axes can be used to quantify body oscillations in the time domain and time frequencies. These measurements are comparable to those obtained with force platforms [10]. In the case of dynamic balance assessment, inertial sensors have been used during tests like the sit-to-stand test, which introduce various additional variables beyond the number of sit-and-stand cycles performed within a specific time, as is customary in clinical testing [26]. The assessment of mobility via the Timed Up and Go test solely relies on the time it takes to perform [27]. However, the addition of a smartphone to the test can provide measurements of acceleration during the process of getting up and down from a chair, as well as the time taken to walk to and from the chair, and the angular velocity of turns taken during the test [28].

In addition to balance and mobility tests, performing other motor tests on smartphones can also be of interest for evaluating patients with leprosy. The Finger Tapping Test and reaction time test, which indirectly indicate nerve conduction time, can provide valuable information, as nerve conduction time may be delayed in patients with leprosy [29,30].

## Factors to Be Considered When Introducing a Smartphone to Monitor Motor Functionality in Older People With Leprosy

Many experts discussing the implementation of smartphones in health care may initially assume that the proposal involves using the patient’s smartphone to acquire all biological signals. However, for digital health interventions, such as motor assessment of patients with leprosy, it is recommended to use smartphones provided by the health care service. Some issues to consider would be the following: (1) the service can ensure that the device meets the necessary technical requirements and is compatible with the software; (2) few studies have evaluated the reliability of measurements using smartphones in unsupervised motor tests; (3) self-tests using smartphones may not be suitable for potentially fragile patients, as some tests (balance and mobility) require trained personnel to be present to prevent falls; and (4) accessing patient history data for evaluation purposes would be decentralized and challenging for managers. The latter is also a factor that does not indicate that the health care professional’s smartphone should be used.

Few smartphone apps include more than one motor test. Most of the apps validated in the literature are still experimental and not available in virtual app stores. Some of the available apps provide files with time series of sensor recordings for offline analyses using computational routines. Furthermore, applications that report the values of the extracted characteristics do not provide normative reference values for interpreting those measurements. Therefore, before any intervention, it is necessary to have a plan for processing and storing the data so that the intervention is not frustrating.

While smartphone-based movement analysis apps offer great promise, these interventions are not without significant challenges. Addressing cost, privacy, digital literacy, regulatory, and systemic barriers will be critical for successful implementation, particularly in low- and middle-income countries (LMICs) and older populations. A well-rounded strategy involving stakeholder collaboration, robust funding mechanisms, and tailored solutions can help overcome these obstacles, ensuring that these tools fulfill their potential for equitable health care delivery.

While smartphones are more affordable than gold-standard equipment, they may still be expensive for underfunded health systems or patients in LMICs. Bulk purchases by governments or institutions might reduce costs, but initial investments could strain already limited budgets. Beyond initial procurement, ongoing costs for repairs, software updates, and network connectivity could be prohibitive. Health systems in LMICs often struggle with basic infrastructure issues (eg, lack of clean water and staff shortages). Allocating resources to digital health tools could be viewed as a lower priority compared to addressing these fundamental needs. Investing in smartphone-based interventions might divert funds from critical areas like vaccine programs, malnutrition initiatives, or essential drug supplies. Governments and stakeholders would need to weigh the benefits of early diagnosis and monitoring of movement disorders against these other pressing needs. Another possibility is looking for other mechanisms for funding such as partnerships with nonprofits or nongovernmental organizations focused on health equity, international aid or grants from organizations like the WHO or international foundations (such as the Gates Foundation), and public-private collaborations where technology companies subsidize costs for social impact.

Storing sensitive health data on cloud servers could expose patients to risks of data breaches or misuse. Many LMICs lack robust data protection laws, creating additional vulnerabilities. Some strategies can be applied to mitigate these vulnerabilities such as the use of encryption for data storage and transmission, storage of data locally on the device where feasible, minimization of cloud reliance, and development and enforcement of clear, transparent privacy policies, thereby ensuring compliance with international standards like General Data Protection Regulation where applicable. Patients need to understand how their data will be used and stored. Consent processes must be clear, culturally appropriate, and account for varying levels of digital literacy.

Many older adults, especially those in LMICs, may lack digital literacy (familiarity with smartphones), have physical barriers (poor vision, tremors, or limited dexterity), distrust in technology, or fear of making mistakes, which might discourage adoption. Developers should consider assistive technologies, such as voice commands or larger, high-contrast displays, and simplified user app interfaces; stakeholders should consider training and support programs for the patients, although they might add to implementation complexity.

The regulatory requirements for medical devices and health apps vary significantly among countries. In LMICs, regulatory frameworks for digital health tools may be underdeveloped or nonexistent. In contrast, high-income countries might impose rigorous standards, thus delaying deployment. Early engagement of local regulatory bodies is necessary to clarify requirements and ensure compliance. Some cultures may have reservations about adopting digital health tools due to mistrust of external technology or ethical concerns. Tailored community engagement and education are crucial.

Despite these challenges, we believe that the introduction of a smartphone for motor assessment as part of a digital health intervention holds potential benefits for all stakeholders involved in caring for patients with leprosy. Health professionals would be able to access objective data on various functional abilities for comparative assessment of therapy effectiveness. Health managers could offer personalized services based on functional characteristics of patient demand and plan health policies tailored to the population served by the service. Patients stand to benefit from enhanced health monitoring, as explained above.

## Abbreviations

LMIC: low- and middle-income country
WHO: World Health Organization
